# A dissonant scale: stress recognition in the SAQ

**DOI:** 10.1186/1756-0500-6-302

**Published:** 2013-07-31

**Authors:** Jennifer A Taylor, Ravi Pandian

**Affiliations:** 1 Department of Environmental and Occupational Health, Drexel University School of Public Health, Philadelphia, PA, USA

**Keywords:** Safety attitudes questionnaire, Stress recognition, Factor analysis

## Abstract

**Background:**

Our previous analyses using the Stress Recognition subscale of the Safety Attitudes Questionnaire (SAQ) resulted in significant effect estimates with equally opposing explanations. We suspected construct validity issues and investigated such using our own data and correlation matrices of previous published studies.

**Methods:**

The correlation matrices for each of the SAQ subscales from two previous studies by Speroff and Taylor were replicated and compared. The SAS Proc Factor procedure and the PRIORS = SMC option were used to perform Common Factor Analysis.

**Results:**

The correlation matrices of both studies were very similar. Teamwork, Safety Climate, Job Satisfaction, Perceptions of Management and Working Conditions were well-correlated. The correlations ranged from 0.53 to 0.76. For Stress Recognition correlations ranged from -0.15 to 0.03. Common Factor Analysis confirmed the isolation of Stress Recognition. CFA returned a strong one-factor model that explained virtually all of the communal variance. Stress Recognition loaded poorly on this factor in both instances, and the CFA indicated that 96.4-100.0% of the variance associated with Stress Recognition was unique to that subscale, and not shared with the other 5 subscales.

**Conclusions:**

We conclude that the Stress Recognition subscale does not fit into the overall safety climate construct the SAQ intended to reflect. We recommend that this domain be omitted from overall safety climate scale score calculations, and clearly identified as an important yet distinct organizational construct. We suggest that this subscale be investigated for its true meaning, characterized as such, and findings conveyed to SAQ end users. We make no argument against Stress Recognition as an important organizational metric, rather we suggest that as a stand-alone construct its current packaging within the SAQ may be misleading for those intent on intervention development and evaluation in healthcare settings if they interpret Stress Recognition results as emblematic of safety climate.

## Background

Our previous work with the SAQ raised validity concerns regarding its Stress Recognition subscale [1,2]. This paper provides an update to our investigation of this domain. The Safety Attitudes Questionnaire (SAQ) is one of the most highly validated hospital climate assessment instruments in use today [3]. The instrument elicits frontline healthcare workers’ perceptions of their organization’s safety culture at the level of the clinical area on which they work. Subscales within the SAQ include Teamwork, Safety Climate, Job Satisfaction, Perceptions of Management, Working Conditions, and Stress Recognition. The SAQ has been used to assess opportunities for quality improvement in obstetrical settings [4], intensive care units [5,6], within single institutions [7-9], in multicentre studies [10-12], children’s hospitals [13], the Veteran’s Administration [14] and increasingly in international settings [8,15]. The SAQ has been well-described [16-18].

In 2011, we published a paper showing a positive relationship between increasing scores on subscales of the Safety Attitudes Questionnaire and associations with decreasing nurse and patient injuries [1]. In the results of that paper, we did not report one of the six subscales of the SAQ: Stress Recognition. We hypothesized that there might be construct validity concerns because Stress Recognition acted very differently from the other SAQ subscales. The results of our multilevel logistic regression showed a threefold increase in the odds of nurse injury with every 10 unit increase in stress recognition among nurses in a Level I Trauma Center. We saw similar relationships with medical errors, whereby increasing Stress Recognition was associated with a 1.5 to 3-fold increase in the odds of patient falls, medication errors, and decubitus ulcers [1,2]. The stress recognition subscale of the SAQ was intended to measure frontline healthcare workers’ understanding that working in a highly stressful environment could put them in adverse conditions that might result in harm to their patients. Given our understanding of how the subscale was intended to work, we could not interpret our results conclusively and did not include them in our previous manuscript. We mentioned at the time that we were exploring this subscale for construct validity issues and present the results of such herein.

## Methods

Analysis began with reconstruction of the correlation matrices for each of the SAQ subscales. Only two published studies reported correlations for the entire dataset: Speroff [12] and Taylor [1]. Both matrices were reviewed, and the relationships amongst the subscales were examined. Common Factor Analysis (CFA) was then utilized to identify the number of latent traits underlying the six subscales, and to determine the relationship of the subscales with the identified trait(s). To execute the CFA, the SAS (version 9.2) Proc Factor procedure was used in conjunction with the PRIORS = SMC option (which utilizes squared multiple correlations to estimate shared communal variance). Given that a one factor (i.e. one dimensional) model was returned, the rotation of factor loadings could not be conducted.

## Results

Table 1 shows the items comprising the Stress Recognition subscale compared with those of the other 5 SAQ subscales. From a face validity perspective, the Stress Recognition items elicit an individual perspective about abilities (“I am more likely to make errors in tense or hostile situations” [emphasis added]) while the items on the other SAQ subscales elicit perspectives about their work area or broader organizational unit. (“The culture in this clinical area makes it easy to learn from the errors of others” [emphasis added]).

**Table 1:** Comparison of questions in the SAQ by subscale

**SAQ SubScale**	**Survey questions**
Stress recognition	When my workload becomes excessive, my performance is impaired
	I am less effective at work when fatigued
	I am more likely to make errors in tense or hostile situations
	Fatigue impairs my performance during emergency situations (e.g. emergency resuscitation, seizure)
Teamwork	Nurse input is well received in this clinical area
	In this clinical area, it is difficult to speak up if I perceive a problem with patient care
	Disagreements in this clinical area are resolved appropriately (i.e., not who is right, but what is best for the patient)
	I have the support I need from other personnel to care for patients
	It is easy for personnel here to ask questions when there is something that they do not understand
	The physicians and nurses here work together as a well-coordinated team
Safety climate	I would feel safe being treated here as a patient
	Medical errors are handled appropriately in this clinical area
	I know the proper channels to direct questions regarding patient safety in this clinical area
	I receive appropriate feedback about my performance
	In this clinical area, it is difficult to discuss errors
	I am encouraged by my colleagues to report any patient safety concerns I may have
	The culture in this clinical area makes it easy to learn from the errors of others
Morale or job satisfaction	I like my job
	Working here is like being part of a large family
	This is a good place to work
	I am proud to work in this clinical area
	Morale in this clinical area is high
Perceptions of hospital management	Hospital management supports my daily efforts
	Hospital management doesn't knowingly compromise the patient safety
	Hospital management is doing a good job
	Problem personnel are dealt with constructively by our hospital management
	I get adequate, timely information about events that might affect my work from hospital management
Working conditions	The levels of staffing in this clinical area are sufficient to handle the number of patients
	This hospital does a good job of training new personnel
	All the necessary information for diagnostic and therapeutic decisions is routinely available to me
	Trainees in my discipline are adequately supervised

The similarities between the correlation matrices of Speroff and Taylor were immediately apparent. Teamwork, Safety Climate, Job Satisfaction, Perceptions of Management and Working Conditions were well-correlated. In Speroff, the correlations ranged from 0.66 to 0.76 (Table 2). For Taylor, the range was from 0.53 to 0.73 (Table 3). Stress Recognition was isolated in both results. For Speroff, Stress Recognition correlations ranged from -0.15 to -0.17, and for Taylor, they ranged from -0.06 to 0.03.

**Table 2:** SAQ dimensions, Speroff (2010), descriptive statistics and correlations

	**Teamwork**	**Safety climate**	**Job satisfaction**	**Working conditions**	**Perceptions of mgmt.**	**Stress recognition**
**Mean**	3.75	3.79	3.77	3.45	3.3	**3.68**
**STD**	0.66	0.6	0.73	0.76	0.87	**0.74**
**N**	1406	1406	1406	1406	1406	**1406**
**Teamwork**	1	0.8	0.76	0.69	0.67	**-0.15**
**Safety climate**	0.8	1	0.75	0.7	0.66	**-0.15**
**Job satisfaction**	0.76	0.75	1	0.68	0.73	**-0.17**
**Working conditions**	0.69	0.7	0.68	1	0.7	**-0.16**
**Perceptions of Mgmt.**	0.67	0.66	0.73	0.7	1	**-0.16**
**Stress recognition**	**-0.15**	**-0.15**	**-0.17**	**-0.16**	**-0.16**	**1**

**Table 3:** SAQ dimensions, Taylor (2011), descriptive statistics and correlations

	**Teamwork**	**Safety climate**	**Job satisfaction**	**Working conditions**	**Perceptions of mgmt.**	**Stress recognition**
**Mean**	77.35	76.67	71.86	71.46	61.38	**73.05**
**STD**	18.98	17.21	24.16	20.00	23.99	**22.48**
**N**	902	902	902	902	902	**902**
**Teamwork**	1	0.73	0.67	0.58	0.59	**-0.05**
**Safety climate**	0.73	1	0.64	0.58	0.62	**-0.06**
**Job satisfaction**	0.67	0.64	1	0.53	0.63	**0.00**
**Working conditions**	0.58	0.58	0.53	1	0.63	**-0.04**
**Perceptions of Mgmt.**	0.59	0.62	0.63	0.63	1	**0.03**
**Stress recognition**	**-0.05**	**-0.06**	**0.00**	**-0.04**	**0.03**	**1**

**Figure 1:**
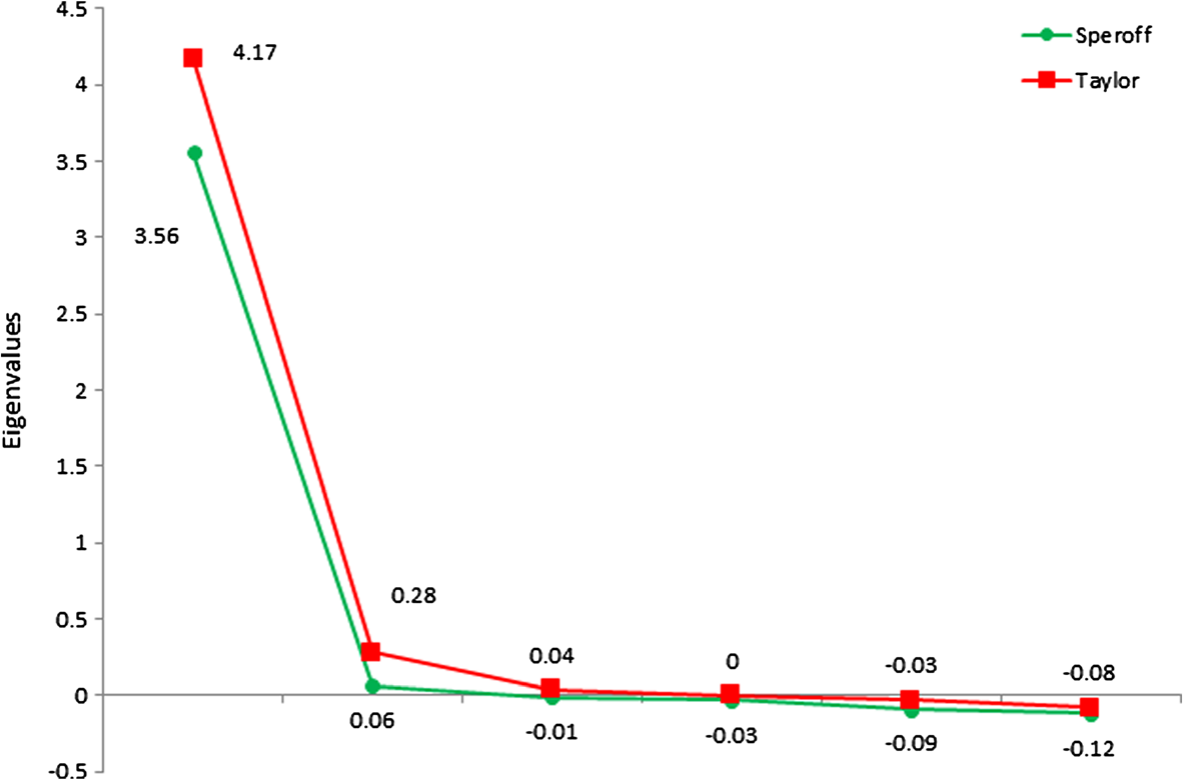
Scree plot of eigenvalues for Speroff and Taylor.

Exploratory Common Factor Analysis confirmed that 96% + of the variance in the Stress Recognition subscale was unique to that scale and not “in common” with the others. That fact demonstrates the isolation of the scale. The number of the factors used in our model was determined by the eigenvalues themselves as evidenced in the skree plot for both Taylor and Speroff (Figure 1). For both Speroff and Taylor, CFA returned a strong one-factor model that explained virtually all of the communal variance (Table 4). Stress Recognition loaded poorly on this factor in both instances, and the CFA indicated that 96.4% (Speroff) and 100.0% (Taylor) of the variance associated with Stress Recognition was unique to that subscale, and not shared with the other 5 components.

**Table 4:** Loadings for Speroff and Taylor’s one factor model

	**Speroff factor loadings**	**Taylor factor loadings**
**Teamwork**	0.865	0.818
**Safety climate**	0.862	0.815
**Job satisfaction**	0.861	0.777
**Working conditions**	0.805	0.716
**Perceptions of mgmt.**	0.805	0.77
**Stress recognition**	**-0.188**	**-0.032**

We were precluded from conducting a similar analysis with the Sexton data. Sexton reported between and within-area correlations, but not correlations for the entire dataset. Similarly, he reported descriptive statistics for geographical sub-samples, but not the sample as a whole. Thus we did not have access to the necessary data.

## Discussion

We focused on the psychometric validation of the SAQ to determine whether Stress Recognition is related to the overall safety climate construct. As evidenced by its weak fit with the other subscales of the SAQ, we conclude that the Stress Recognition subscale does not fit into the overall safety climate construct measured by the SAQ.

These results were confirmed by the work of Gallego et al. As explanation, Gallego suggest that “Stress Recognition items differ from those in other SAQ scales in that they assess self-behaviour while other SAQ scales focus mainly on behaviours of others in the respondent’s workplace …” and that “Stress Recognition scales … seem likely to remain relatively constant …” [19]. Pettker et al. administered the SAQ before and after a obstetrics patient safety program, and observed that while the other scales changed in a statistically significant manner, Stress Recognition did not [20]. While not definitive proof, such longitudinal testing would offer a way to evaluate the circumstances in which Stress Recognition changes relative to the other scales, and could either confirm or deny Gallego’s self-behavior versus others-workplace-behavior hypothesis above.

This finding is also supported by the previous work of Speroff who in reflection on their own correlations using the SAQ in 1,406 respondents stated that "the stress recognition items do not contribute positively towards the construct of safety climate as intended and should be excluded from the SAQ" [12]. We believe that the inclusion of the Stress Recognition subscale in the SAQ does not affect its overall reliability. However, we have strong concerns that the stress recognition subscale of the SAQ affects its validity. We recommend that this domain be omitted from overall safety climate scale score calculations, and clearly identified as a distinct construct.

The Stress Recognition subscale of the SAQ was intended to measure frontline healthcare workers’ understanding that working in a highly stressful environment could put them in adverse conditions that might result in harm to their patients. Stress Recognition is defined as “the extent to which individuals acknowledge personal vulnerability to stressors such as fatigue, personal problems, and emergencies situations” [21]. Furthermore, “Stress recognition may be enhanced or jeopardized by organizational practices such as scheduling, supervision and staffing levels” [22].

As constructed, this subscale is a measure of organizational buy-in: instead of blaming oneself, workers understand that working conditions create stressors which make them unable to do their jobs as well as they would normally expect. When we attempted to interpret our findings regarding the very significant increased odds of nurse and patient injury associated with increasing Stress Recognition, we came to two equally opposing explanations. The first was that the organization has been effective in its efforts to teach nurses that environmental stressors can lead to unsafe conditions, so nurses in turn report more occupational and patient injuries than nurses who have not. This type of reporting would be reflective of workers who have bought into the organizational effort to affirm that their concerns they would be heard and may garner additional resources to help ameliorate problems they face (e.g., low staffing levels, high turnover).

The alternative explanation was that when nurses were responding to these SAQ Stress Recognition items that they actually read them as indicative of measuring their stress *level* at the time. While this is not how the subscale was designed, it may in fact be how nurses are interpreting the items. In discussions with the SAQ developer, the Stress Recognition domain of the SAQ operates somewhat differently than the other domains. It represents the individual attitudes of the respondents rather than a consensus among those people working on the unit (Bryan Sexton, personal communication). Since the nurse injuries described in our 2012 publication were voluntary reports to the Hospital’s Occupational Health Department, the effect of increased stress recognition may represent an increased awareness of the importance of reporting injuries due either to heightened awareness of stress or because of a past stress-related experience. Our analysis herein did not address these competing hypotheses, but future research should investigate how respondents are interpreting the survey items.

We consider the poor fit of Stress Recognition within the SAQ as important because many hospitals and healthcare facilities use the SAQ as a baseline for improvement opportunities. We are concerned that the subscale is measuring something other than safety climate and therefore hospitals initiating interventions to improve stress recognition may not see a difference if they are using interventions specific to safety climate to make change. Conversely, hospitals with active safety climate interventions may not see any change in Stress Recognition on the SAQ because of its lack of association with safety climate.

## Conclusions

We found that the Stress Recognition subscale of the Safety Attitudes Questionnaire does not fit with the overall heuristic of the instrument as designed. The other five subscales are highly correlated with one another, as evidenced by the results of the factor analysis. Stress Recognition is a standalone construct. The intent of the subscale was to capture attitudes that reflected an increased understanding of the role stress plays in the ability to do one's job safely. While the construct may indeed be capturing this exact perspective (and we offer no counterargument to the importance of understanding how stress works in one’s environment), Stress Recognition is a separate and distinct measure of organizational buy-in and is not reflective of safety climate. It may be indicative of fatigue as a precursor to burnout as evidenced in the work of Raftopoulos et. al. [23] Healthcare organizations seeking to improve their stress recognition measurements should be mindful that turning to proven safety climate interventions (e.g., executive walkarounds, teamwork training) may not produce expected results because stress recognition is not a part of safety climate and therefore not sensitive to interventions designed to improve it. Instead, they should review the fatigue and burnout literature to examine potential solutions to the perceptions the SAQ stress recognition domain is measuring in their workforce.

## Competing interests

The authors declare they have no financial or non-financial competing interests.
